# Disparities in COVID-19 fatalities among working Californians

**DOI:** 10.1371/journal.pone.0266058

**Published:** 2022-03-29

**Authors:** Kristin J. Cummings, John Beckman, Matthew Frederick, Robert Harrison, Alyssa Nguyen, Robert Snyder, Elena Chan, Kathryn Gibb, Andrea Rodriguez, Jessie Wong, Erin L. Murray, Seema Jain, Ximena Vergara

**Affiliations:** 1 Occupational Health Branch, California Department of Public Health, Richmond, CA, United States of America; 2 Public Health Institute, Oakland, CA, United States of America; 3 Infectious Diseases Branch, California Department of Public Health, Richmond, CA, United States of America; 4 Immunization Branch, California Department of Public Health, Richmond, CA, United States of America; 5 Heluna Health, City of Industry, CA, United States of America; Freelance Consultant, Myanmar, MYANMAR

## Abstract

**Background:**

Information on U.S. COVID-19 mortality rates by occupation is limited. We aimed to characterize 2020 COVID-19 fatalities among working Californians to inform preventive strategies.

**Methods:**

We identified laboratory-confirmed COVID-19 fatalities with dates of death in 2020 by matching death certificates to the state’s COVID-19 case registry. Working status for decedents aged 18–64 years was determined from state employment records, death certificates, and case registry data and classified as “confirmed working,” “likely working,” or “not working.” We calculated age-adjusted overall and occupation-specific COVID-19 mortality rates using 2019 American Community Survey denominators.

**Results:**

COVID-19 accounted for 8,050 (9.9%) of 81,468 fatalities among Californians 18–64 years old. Of these decedents, 2,486 (30.9%) were matched to state employment records and classified as “confirmed working.” The remainder were classified as “likely working” (n = 4,121 [51.2%]) or “not working” (n = 1,443 [17.9%]) using death certificate and case registry data. Confirmed and likely working COVID-19 decedents were predominantly male (76.3%), Latino (68.7%), and foreign-born (59.6%), with high school or less education (67.9%); 7.8% were Black. The overall age-adjusted COVID-19 mortality rate was 30.0 per 100,000 workers (95% confidence interval [CI], 29.3–30.8). Workers in nine occupational groups had age-adjusted mortality rates higher than this overall rate, including those in farming (78.0; 95% CI, 68.7–88.2); material moving (77.8; 95% CI, 70.2–85.9); construction (62.4; 95% CI, 57.7–67.4); production (60.2; 95% CI, 55.7–65.0); and transportation (57.2; 95% CI, 52.2–62.5) occupations. While occupational differences in mortality were evident across demographic groups, mortality rates were three-fold higher for male compared with female workers and three- to seven-fold higher for Latino and Black workers compared with Asian and White workers.

**Conclusion:**

Californians in manual labor and in-person service occupations experienced disproportionate COVID-19 mortality, with the highest rates observed among male, Latino, and Black workers; these occupational group should be prioritized for prevention.

## Introduction

Following the identification of community transmission of SARS-CoV-2 in California in late February 2020, the state became the first in the nation to issue a stay-at-home order on March 19, 2020 [1–4]. The order identified critical infrastructure sectors, including health, emergency services, food and agriculture, and transportation and logistics, in which Californians could work outside of the home [4, 5]. As a result, at least 4.7 million Californians, or 25% of the entire workforce, continued to work in person with coworkers and/or members of the public [6].

Working-age adults (18–64 years old) account for 72% of confirmed COVID-19 cases and 29% of confirmed COVID-19 deaths in California [7]. Whether conditions in the workplace contributed to these cases and fatalities is important to know, as this information could be used to prioritize preventive interventions to reduce opportunities for workplace transmission, such as respiratory protection or improved ventilation, and to guide testing and vaccination policies. A first step in understanding workplace contributors to COVID-19 in California is to determine if particular occupations are at higher risk of COVID-19 disease and death.

Yet, aside from descriptions of workplace cases and outbreaks [3, 8–10], little is known about COVID-19 morbidity and mortality rates by occupation in the state. A study of deaths that occurred from March to November 2020 among Californians 18–65 years old found relative excess all-cause mortality in specific occupational sectors, suggesting differential impact of COVID-19 on California’s workers [11]. However, the authors used the occupation listed on the death certificate, which assumed that working-age decedents had been actively engaged in employment around the time of death. This standard approach undoubtedly led to misclassification of some unemployed, retired, or otherwise not working decedents as working in a particular sector. Furthermore, this study included causes of death other than COVID-19. These definitions of exposure and outcome could obscure the association between occupation and COVID-19 fatality. Hence, methodologic improvements in the assessment between occupation and COVID-19 fatality are needed.

We aimed to identify high-risk occupations among working Californians in 2020 by examining COVID-19 mortality rates by occupational group, using a novel approach to verify employment. We hypothesized that the mortality rate would vary across occupational groups, with essential workers and others working in-person having higher mortality. To increase specificity, we focused on decedents who were working at the time of death and on laboratory-confirmed COVID-19 deaths. We also calculated a Prevention Index for detailed occupations that takes into account both the number of deaths and the mortality rate for a particular occupation, which can be used to prioritize preventive interventions.

## Methods

### Data sources

We used three data sources to identify working Californians who died of COVID-19 in 2020. These were: 1) the Electronic Death Registration System (EDRS), which contains California’s death certificates; 2) the state’s COVID-19 case registry, comprising all laboratory-confirmed COVID-19 cases among California residents reported to the California Department of Public Health (CDPH) by local health jurisdictions; and 3) Employment Development Department (EDD) records derived from quarterly tax reports submitted to the EDD by employers subject to the state’s Unemployment Insurance laws and by federal agencies in California subject to the Unemployment Compensation for Federal Employees program [12].

We used local health jurisdiction determinations included in the COVID-19 case registry as of April 15, 2021 to define COVID-19 decedents; all other deaths were considered non-COVID decedents. Case registry records of COVID-19 decedents were probabilistically matched to an EDRS dynamic file dated April 21, 2021 using first, middle, and last names; date of birth; date of death; zip code; city; county; and cause of death. The final dataset was restricted to decedents who were 18–64 years old at death. Age and other demographic characteristics were derived from the EDRS records.

We used EDD records to examine decedents’ 2020 employment by calendar quarter. Exact matching between EDRS and EDD records used social security number from the death certificates. Members of the armed forces, the self-employed, proprietors, domestic workers, unpaid family workers, and railroad workers are not covered by the unemployment insurance systems noted above and are therefore excluded from the EDD data [12]. In addition, workers in the “underground economy,” defined by EDD as “individuals and businesses that deal in cash and/or use other schemes to conceal their activities…from government” are not captured by EDD data [13].

### Working status classification

We classified decedents as *confirmed working* at the time of infection if EDD records indicated that the decedent was employed during the quarter of death or the previous quarter. Decedents were classified as *not working* at the time of infection and excluded if the EDRS or COVID-19 case registry records indicated that the decedent was unemployed, retired, incarcerated, not paid, a student, or a homemaker. Because EDD records cover only a subset of the workforce, a decedent who did not meet criteria for *confirmed working* or *not working* was classified as *likely working* at the time of infection and included.

## Statistical analyses

U.S. Census Occupation Codes (2010) were assigned to confirmed working and likely working decedents’ death certificate free text for “usual occupation” using a machine learning-based system, the National Institute for Occupational Safety and Health (NIOSH) Industry and Occupation Computerized Coding System (NIOCCS) [14]. We conducted manual coding when NIOCCS reported less than 90% confidence of accuracy for an autocode.

The U.S. Census Bureau’s 2019 American Community Survey (ACS) Public Use Microdata Sample files for California were extracted by detailed occupation, age, sex, and race/ethnicity [15]. We calculated crude overall, occupational group-specific, and detailed occupation-specific COVID-19 mortality rates by combining confirmed and likely working decedents for numerators and using ACS data to generate denominators. Rates were calculated for five or more deaths per occupational group, detailed occupation, or by subgroup. Deaths were aggregated at the occupational group level: 1) overall, 2) by sex, and 3) by race/ethnicity to calculate age-adjusted rates. Direct age standardization to the ACS employment data was performed using five age groups: 18–29, 30–39, 40–49, 50–59, and 60–64 years. Combined age-, sex- and race/ethnicity-standardization was not possible due to sparse data.

We calculated a Prevention Index for each detailed occupation by taking the average of the rank orders of fatality counts and crude mortality rates [16]. For instance, a detailed occupation with a rank order of 2 for fatality count and 10 for crude mortality rate would have a Prevention Index rank order of 6.

Statistical analyses were conducted using SAS software V.9.4 (SAS Institute, Inc, Cary, North Carolina, USA) and R Studio Version 4.0.2 (R Studio, PBC, Boston, Massachusetts, USA). Rates were calculated using the R epitools package.

### Ethical considerations

The California Health and Human Services Agency’s Committee for the Protection of Human Subjects determined that this project was exempt from review because the activities involved public health practice/surveillance, not research. Informed consent for use of identifiable information by a public health agency for public health practice/surveillance is not required.

## Results

### 2020 California decedents

**Fig 1:**
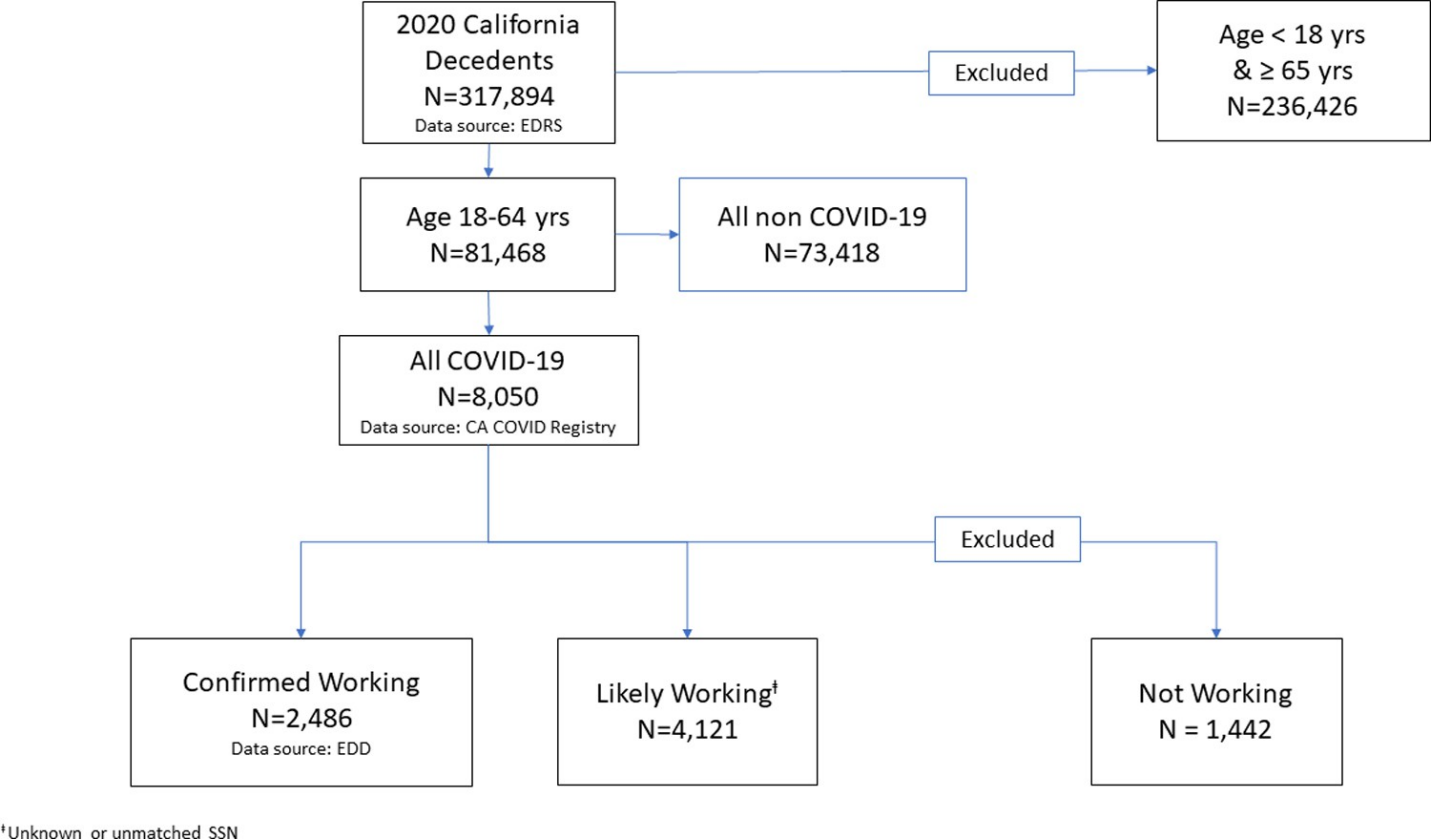
Identification of California’s 2020 COVID-19 decedents ages 18–64 years and classification according to working status. Laboratory-confirmed fatal COVID-19 cases were reported by local health jurisdictions to the California Department of Public Health. Working status was classified according to state employment records and information available on death certificates and in California’s COVID-19 case registry. Abbreviations: EDRS, Electronic Death Registration System; EDD, Employment Development Department.

**Fig 2:**
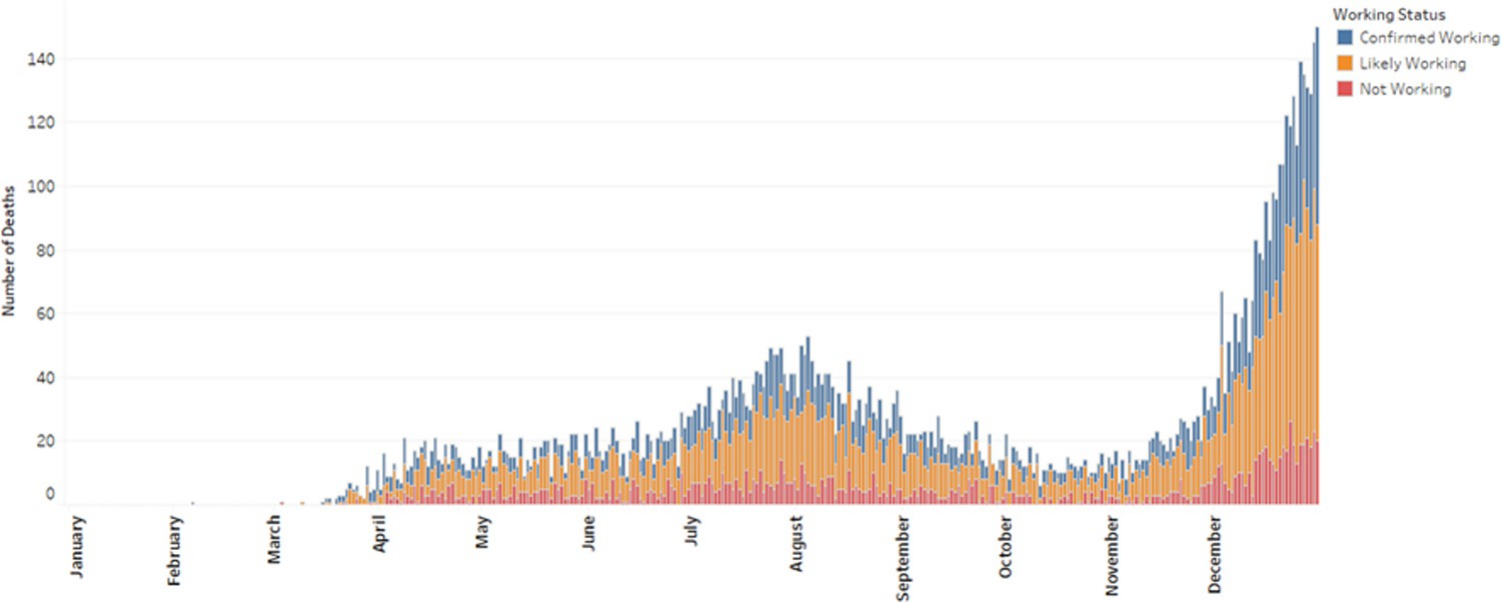
COVID-19 deaths among decedents ages 18–64 years, by day and decedents’ working status, California, 2020. Laboratory-confirmed fatal COVID-19 cases were reported by local health jurisdictions to the California Department of Public Health. Working status was classified according to state employment records and information available on death certificates and in California’s COVID-19 case registry.

Among 317,894 people who died in California in 2020, we identified 81,468 (25.6%) who were 18–64 years old at the time of death, including 8,050 (9.9%) COVID-19 decedents (Fig 1). Among the COVID-19 decedents, 2,486 (30.9%) were classified as confirmed working, 4,121 (51.2%) as likely working, and 1,443 (17.9%) as not working. The number of COVID-19 decedents by day reflected the state’s overall epidemic curve [7], with the proportions by working status relatively stable throughout 2020 (Fig 2).

Compared to non-COVID-19 decedents, all COVID-19 decedents, regardless of working status, more often were older, male, Latino, and foreign-born and had lower education levels (Table 1). A total of 7,214 (9.8%) non-COVID-19 decedents and 1,536 (19.1%) COVID-19 decedents lacked a valid social security number on the death certificate, including 1,222 (29.7%) likely working COVID-19 decedents.

**Table 1:** Non-COVID-19 fatalities and COVID-19 fatalities overall and by work status among decedents ages 18–64 years, California, 2020.

	Non-COVID-19 Deaths	COVID-19 Deaths				
			Working Status			
Characteristic, No. (%)	All	All	Confirmed	Likely	Confirmed + Likely	Not Working
No.	73418	8050	2486	4121	6607	1443
Age, y						
18–29	6543 (8.9%)	200 (2.5%)	79 (3.2%)	66 (1.6%)	145 (2.2%)	55 (3.8%)
30–39	8292 (11.3%)	570 (7.1%)	200 (8.0%)	268 (6.5%)	468 (7.1%)	102 (7.1%)
40–49	11746 (16.0%)	1367 (17.0%)	415 (16.7%)	727 (17.6%)	1142 (17.3%)	225 (15.6%)
50–59	25657 (34.9%)	3221 (40.0%)	1062 (42.7%)	1620 (39.3%)	2682 (40.6%)	539 (37.4%)
60–64	21180 (28.8%)	2692 (33.4%)	730 (29.4%)	1440 (34.9%)	2170 (32.8%)	522 (36.2%)
Male sex	48159 (65.6%)	5616 (69.8%)	1849 (74.4%)	3190 (77.4%)	5039 (76.3%)	577 (40.0%)
Race						
Asian	5927 (8.1%)	577 (7.2%)	293 (11.8%)	216 (5.2%)	509 (7.7%)	68 (4.7%)
Black	8961 (12.2%)	609 (7.6%)	173 (7.0%)	342 (8.3%)	515 (7.8%)	94 (6.5%)
Latino	23687 (32.3%)	5520 (68.6%)	1691 (68.0%)	2846 (69.1%)	4537 (68.7%)	983 (68.1%)
White	31745 (43.2%)	1144 (14.2%)	278 (11.2%)	607 (14.7%)	885 (13.4%)	259 (17.9%)
Other^a^ or Unknown	3098 (4.2%)	200 (2.4%)	51 (2.1%)	110 (2.7%)	161 (2.5%)	39 (2.8%)
Non-US birth country^b^	18868 (25.7%)	4678 (58.1%)	1444 (58.1%)	2493 (60.5%)	3937 (59.6%)	741 (51.4%)
No valid social security number	7214 (9.8%)	1536 (19.1%)	0 (0%)	1222 (29.7%)	1222 (18.5%)	314 (21.8%)
Education^c^						
Less than high school	14124 (19.2%)	2991 (37.2%)	709 (28.5%)	1638 (39.7%)	2347 (35.5%)	644 (44.6%)
High school	28573 (38.9%)	2653 (33.0%)	800 (32.2%)	1339 (32.5%)	2139 (32.4%)	514 (35.6%)
Some college or Associate degree	17031 (23.2%)	1402 (17.4%)	603 (24.3%)	635 (15.4%)	1238 (18.7%)	164 (11.4%)
College degree or more	10730 (14.6%)	703 (8.7%)	351 (14.1%)	292 (7.1%)	643 (9.7%)	60 (4.2%)

^a^Includes Multi-Race, Native Hawaiian and Other Pacific Islander, American Indian, Other.

^b^Excludes missing: 2.4% for non-COVID-19 decedents and 1.9% for COVID-19 decedents.

^c^Excludes unknowns.

Compared to not working COVID-19 decedents, confirmed and likely working COVID-19 decedents more often were male and foreign-born and had higher education levels (Table 1). Compared to likely working decedents, confirmed working decedents more often were Asian and had higher education levels.

### COVID-19 mortality rates by occupational group

**Fig 3:**
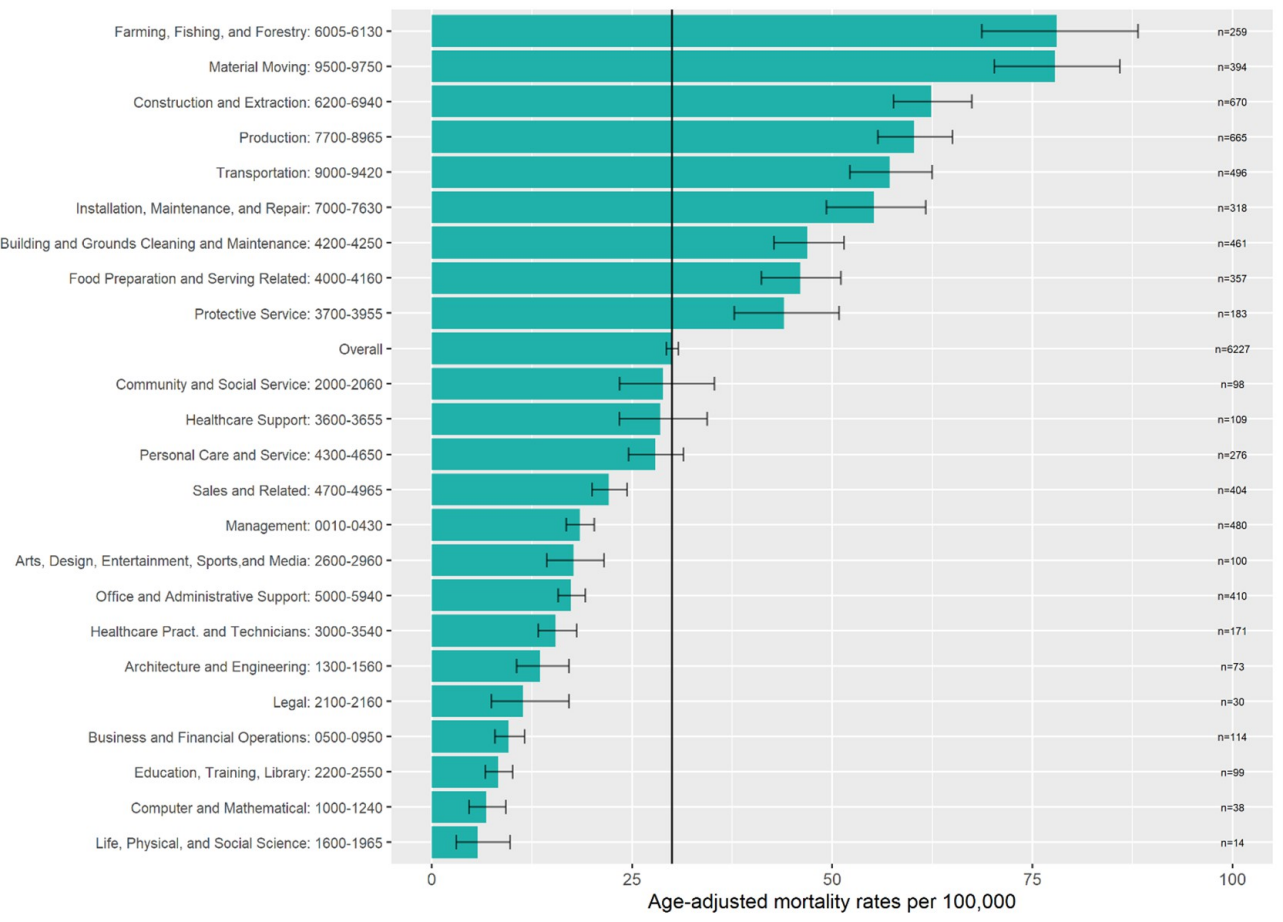
Age-adjusted COVID-19 mortality rates among workers ages 18–64 Years, California, 2020, overall and for all occupational groups. Information on occupations included within each occupational group is available from the U.S. Census Bureau (https://www.bls.gov/cps/cenocc2010.htm).

The overall age-adjusted COVID-19 mortality rate was 30.0 deaths per 100,000 workers (95% CI, 29.3–30.8) (Fig 3). Nine occupational groups had mortality rates higher than this overall rate: farming, fishing, and forestry (78.0 per 100,000; 95% CI, 68.7–88.2); material moving (77.8; 95% CI, 70.2–85.9); construction and extraction (62.4; 95% CI, 57.7–67.4); production (60.2; 95% CI, 55.7–65.0); transportation (57.2; 95% CI, 52.2–62.5); installation, maintenance, and repair (55.2; 95% CI, 49.3–61.7); building and grounds cleaning and maintenance (46.9; 95% CI, 42.7–51.5); food preparation and serving related (46.0; 95% CI, 41.2–51.1); and protective service (44.0; 95% CI, 37.8–50.9).

**Fig 4:**
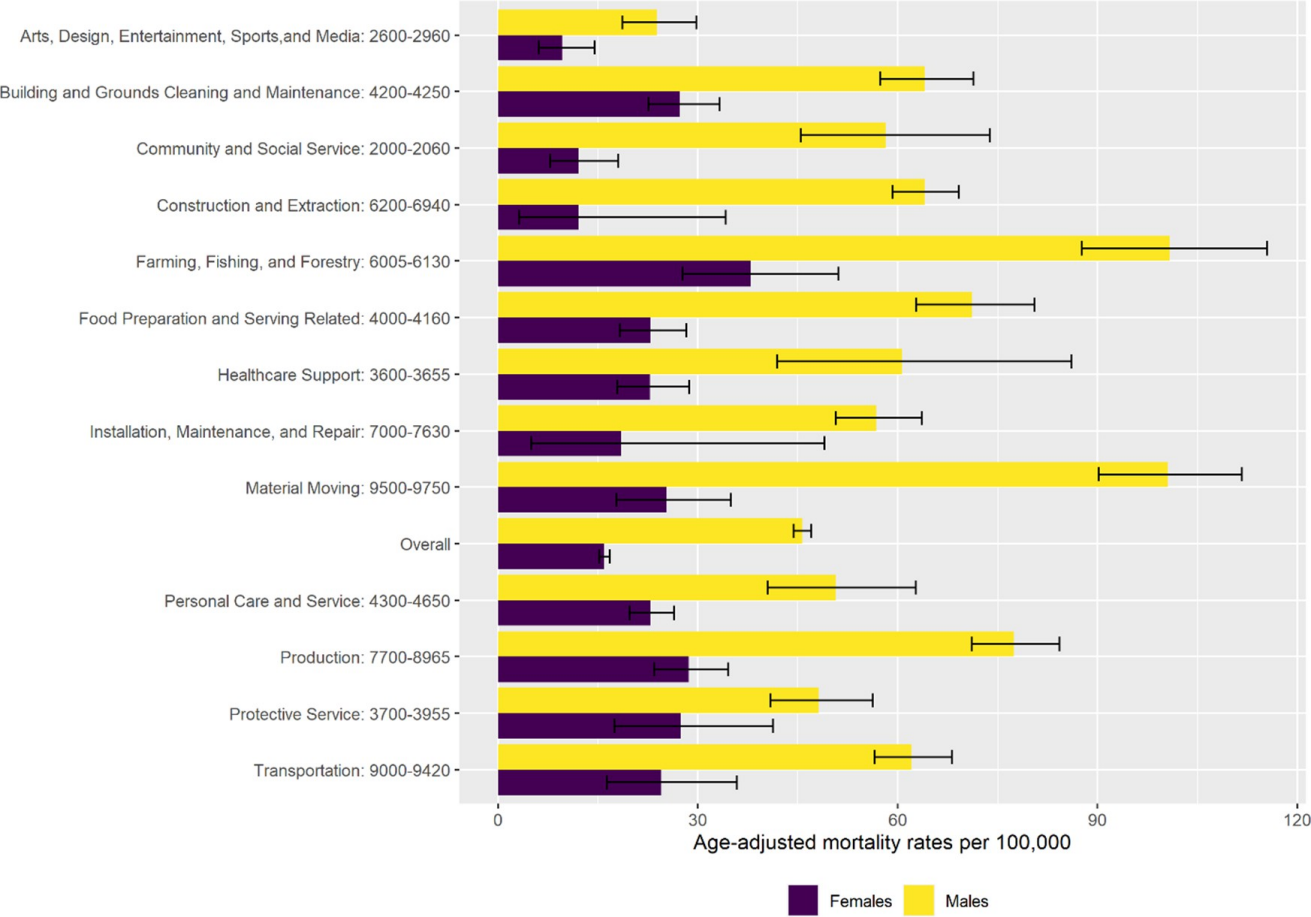
Age-adjusted COVID-19 mortality rates among workers ages 18–64 Years, California, 2020, overall and for select occupational groups, by sex. Information on occupations included within each occupational group is available from the U.S. Census Bureau (https://www.bls.gov/cps/cenocc2010.htm).

The overall age-adjusted COVID-19 mortality rate for male workers (45.7 per 100,000; 95% CI, 44.4–47.0) was nearly three-fold higher than for female workers (15.9; 95% CI, 15.2–16.8) (Fig 4). This pattern was evident for occupation-specific rates as well. Nonetheless, higher-mortality occupational groups were evident for female workers. For instance, female workers in farming, fishing, and forestry had a mortality rate (38.0; 95% CI 27.7–51.1) that was more than twice the rate of female workers overall.

**Fig 5:**
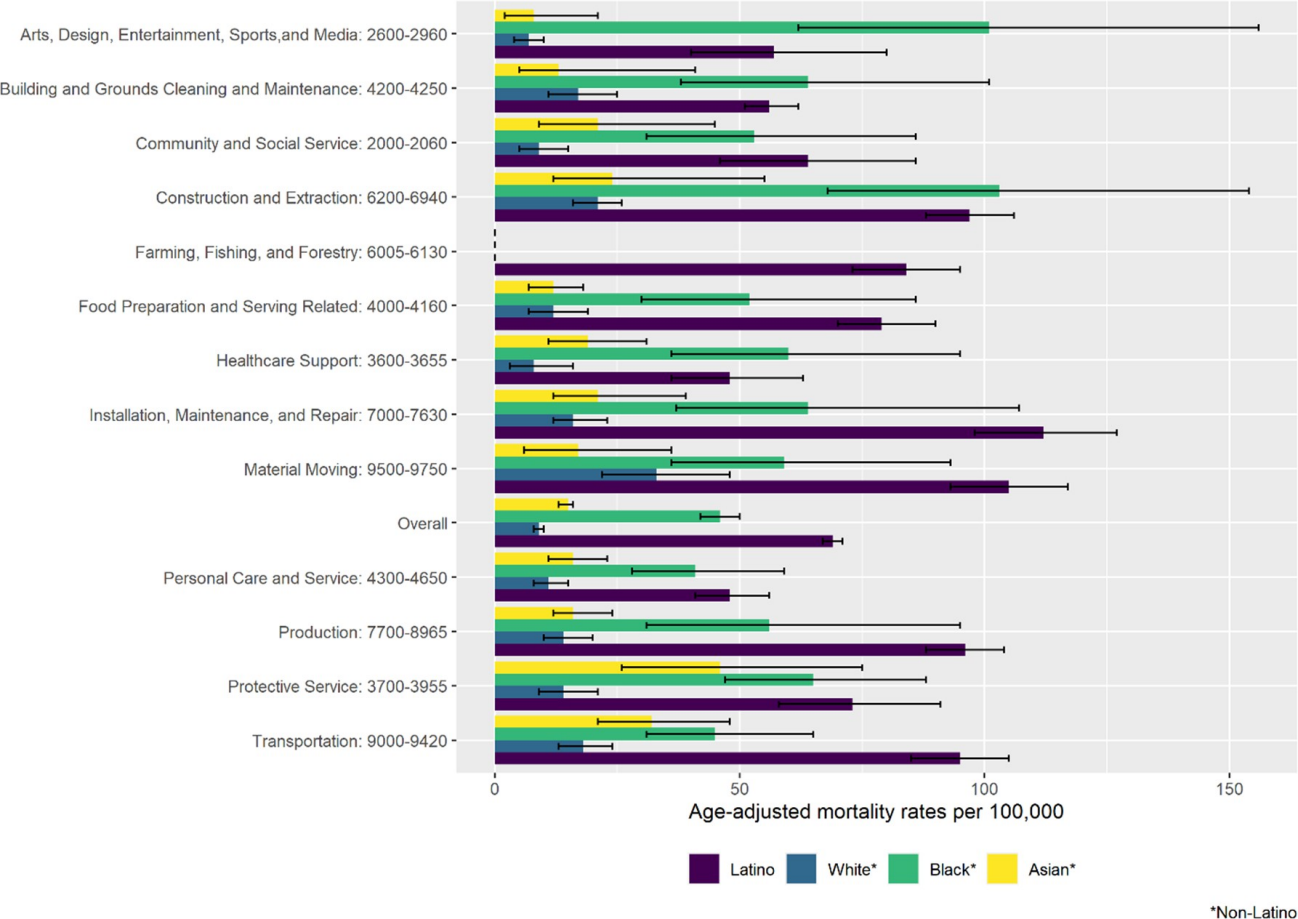
Age-adjusted COVID-19 mortality rates among workers ages 18–64 Years, California, 2020, overall and for select occupational groups, by race/ethnicity. Information on occupations included within each occupational group is available from the U.S. Census Bureau (https://www.bls.gov/cps/cenocc2010.htm).

Overall age-adjusted COVID-19 mortality rates were three- to seven-fold higher for Latino (69.1 per 100,000; 95% CI 67.1.-71.2) and Black (46.4; 95% CI, 42.5–50.6) workers than for Asian (15.0; 95% CI, 13.7–16.4) and White (9.5; 95% CI, 8.9–10.2) workers; similar patterns were observed for occupation-specific rates (Fig 5). Mortality rates were highest for Latino workers in installation, maintenance, and repair (112.1 per 100,000; 95% CI, 98.2–127.4); material moving (105.1; 95% CI, 93.8–117.4); and construction (97.1; 95% CI 88.4–106.6) occupations, and for Black workers in construction and extraction (103.9; 95% CI, 68.1–154.6); arts, design, entertainment, sports, and media (101.2; 95% CI, 62.6–154.6), and protective services (65.4; 95% CI, 47.2–88.6) occupations. Despite lower mortality rates among Asian and White workers compared to Latino and Black workers, higher-mortality occupational groups were evident for Asian and White workers. For instance, Asian workers in protective services occupations (46.5; 95% CI, 26.5–75.9) had a mortality rate that was three-fold higher than the rate for Asian workers overall; the rate for White workers in material moving occupations (33.6; 95% CI, 22.6–48.5) was more than three times higher than the rate for White workers overall.

Age-adjusted COVID-19 mortality rates for healthcare occupational groups were not elevated overall (Fig 3). For healthcare support occupations, male (60.7; 95% CI, 41.9–86.1), Black (60.4; 95% CI, 36.2–95.6), and Latino (48.2; 95% CI, 36.4–63.1) workers had mortality rates higher than the overall rate (28.5; 95% CI, 23.4–34.4); female (22.8; 95% CI, 17.9–28.7), Asian (19.6; 95% CI, 11.5–31.6), and White (8.4; 95% CI, 3.8–16.6) workers had lower rates (Figs 4 and 5). For healthcare practitioner and technician occupations, male (23.3; 95% CI, 18.3–29.4), Latino (35.6; 95% CI, 26.4–47.3), Black (24.4; 95% CI, 14.4–41.6), and Asian (21.0; 95% CI, 16.1–27.3) workers had mortality rates higher than the overall rate (15.5; 95% CI, 13.3–18.1); female (12.2; 95% CI, 9.9–15.0) and White (5.2; 95% CI, 3.5–7.9) workers had lower rates.

### Detailed occupations

The detailed occupation that ranked highest on the Prevention Index was sewing machine operators, followed by construction laborers and automotive service technicians and mechanics (Table 2). The ratio of likely working to confirmed working status among decedents in the top 20 detailed occupations ranged from 0.5 for bus drivers to 6.4 for sewing machine operators, with an average of 2.5.

**Table 2:** Top 20 detailed occupations ranked by mortality prevention index among workers ages 18–64 years, California, 2020.

	Deaths by Working Status, No.			Employment	Crude Rate^a^	PI Rank	Likely: Confirmed^b^
Detailed Occupation (2010 Census Code)	Confirmed	Likely	Total				
Sewing machine operators (8320)	11	70	81	34906	232	1	6.4
Construction laborers (6260)	66	277	343	331577	103	2	4.2
Automotive service technicians and mechanics (7200)	24	92	116	110061	105	3	3.8
Driver/sales workers and truck drivers (9130)	139	227	366	439063	83	4	1.6
Farmers, ranchers, and other agricultural managers (0205)	21	29	50	40803	123	5	1.4
Chefs and head cooks (4000)	21	60	81	84574	96	6	2.9
Industrial truck and tractor operators (9600)	40	41	81	83574	97	7	1.0
Miscellaneous agricultural workers (6050)	82	145	227	296595	77	8	1.8
Laborers and freight, stock, and material movers, hand (9620)	99	147	246	335476	73	9	1.5
Security guards and gaming surveillance officers (3930)	58	73	131	167976	78	10	1.3
Machinists (8030)	16	25	41	36466	112	11	1.6
Clergy (2040)	5	31	36	31718	114	12	6.2
Grounds maintenance workers (4250)	33	104	137	199603	69	13	3.2
Janitors and building cleaners (4220)	84	102	186	300067	62	14	1.2
Welding, soldering, and brazing workers (8140)	11	35	46	57444	80	15	3.2
First-line supervisors of housekeeping and janitorial workers (4200)	12	18	30	27015	111	16	1.5
Supervisors of transportation and material moving workers (9000)	19	12	31	29719	104	17	0.6
Bakers (7800)	10	24	34	37374	91	18	2.4
Painting workers (8810)	5	18	23	19675	117	19	3.6
Bus drivers (9120)	26	13	39	52137	75	20	0.5

Abbreviation: PI, prevention index

^a^Crude rate per 100,000 workers.

^b^Ratio of likely working to confirmed working decedents within detailed occupation.

## Discussion

We found that COVID-19 accounted for 10% of all deaths among working-age Californians in 2020. This burden was unevenly distributed across the state’s population, with COVID-19 and non-COVID-19 decedents ages 18–64 years differing systematically in terms of age, sex, race/ethnicity, country of birth, education level, and possession of a social security number. Among those COVID-19 decedents identified as confirmed or likely working at the time of infection, most were male, Latino, and foreign-born, with a high school education or less. The highest age-adjusted COVID-19 mortality rates were in occupational groups in which work is typically carried out in person: farming, material moving, construction, production, transportation, installation, cleaning, and food service.

Our finding of higher COVID-19 mortality for these occupational groups is consistent with prior studies among Californians and other populations. A study of excess all-cause mortality among Californians 18–65 years old during the first nine months of the pandemic found the highest relative and per capita excess mortality in food/agriculture, transportation/logistics, manufacturing, and facilities sectors [11]. Investigation of COVID-19 deaths among people 16–64 years old in Massachusetts from March through July 2020 also documented above-average mortality rates in the occupational groups we identified, except farming [17]. An occupational analysis of 2020 COVID-19 decedents aged 20–64 years in England and Wales found the highest mortality rates in those working in process plants, security, caring personal services, food service, and transportation [18].

We observed that age-adjusted COVID-19 mortality rates were highest for male, Latino, and Black workers, both overall and within specific occupational groups. Similar patterns were noted in the study of COVID-19 mortality in Massachusetts [17]. Our findings reflect a growing body of literature documenting a disproportionate burden of severe and fatal COVID-19 cases among men and racial/ethnic minorities [19–25]. It is important to note that the occupational disparities in COVID-19 fatalities that we found could not be attributed solely to differences in employment by sex or race/ethnicity. Within each demographic subset, there was a gradient of occupational risk, with remarkable consistency in terms of which occupational groups had higher mortality. One exception was the high mortality rate among Black decedents in arts, design, entertainment, sports, and media occupations. These findings, coupled with the temporal consistencies in working status we observed, suggest specific occupational risks for California’s workers and highlight the intersecting contributions of occupation and other socioeconomic factors to COVID-19 health inequities [26–28].

In contrast to Massachusetts, England, and Wales [17, 18], we did not find above-average COVID-19 mortality in healthcare occupations in California. In Massachusetts, healthcare support occupations had the highest age-adjusted mortality rate of all occupational groups at nearly three times the average [17]. In England and Wales, care workers, home carers, nursing auxiliaries and assistants, and nurses were among the occupations with the highest mortality rates [18]. In California, we found that male, Latino, Black, and, in some cases, Asian workers in healthcare occupations had mortality rates that were higher than the overall rates for these occupational groups, but healthcare occupations were not identified for prioritization by the Prevention Index. The reasons for these discrepancies are uncertain, but they could reflect differences in the time course of the pandemic and COVID-19 deaths. Massachusetts and the United Kingdom were impacted early, with peak numbers of daily deaths for 2020 occurring on days in April [29, 30], whereas deaths peaked in California in December (Fig 2). Given the improvements in understanding of disease transmission and access to personal protective equipment that occurred over the course of 2020 [31–36], California may have been better positioned to protect its healthcare workers during the state’s later epidemic peak. In addition, the potential contribution of California’s longstanding Aerosol Transmissible Diseases standard, which requires healthcare employers to have written safety plans, provide personal protective equipment including respirators for protection from novel pathogens (considered under the standard to be potentially airborne), and train employees on safety procedures, merits further inquiry [37]. A recent study found healthcare workers had the highest rate of COVID-19 related complaints to Cal/OSHA during 2020 despite their relatively low mortality, suggesting a high capacity to advocate for better protections that could prevent infection and death among this group [38]. In addition, compared to the occupational groups that we found to have higher mortality rates in California, healthcare workers in California may have benefited from fewer comorbidities and greater access to care once infected.

A unique strength of our study is the use of state employment records to confirm working status of COVID-19 decedents. Death certificates collect information about a decedent’s “usual” occupation, but do not confirm that the decedent was employed at the time of death [39]. Yet the increase in specificity imparted by state employment records comes at a cost of decreased sensitivity, as many workers are excluded from official employment statistics [12]. In particular, we were concerned about the exclusion of workers in the underground economy, such as day laborers and independent contractors, who make up substantial proportions of some California industries and may be more vulnerable on account of socioeconomic disadvantages [40–42]. Thus, we included a “likely working” group of decedents who did not match to state employment records but for whom there was no documentation of not working status. Over 60% of the likely working decedents were foreign-born and nearly a third lacked a valid social security number, suggesting this group included undocumented immigrants who continued to work in person out of economic necessity [43]. Increased opportunities for exposure in the workplace, in crowded housing, and in other community settings; high rates of comorbidities; and limited access to care are all potential contributors to fatal outcomes among this group that merit further study. Notably, among the top 20 detailed occupations identified by the Prevention Index, the ratio of likely working to confirmed working decedents (2.5) exceeded the ratio for all 6,607 working decedents (1.7).

Despite our use of state employment records to confirm employment, our analyses were not designed to assess a causal relationship between employment and COVID-19 infection and death. The COVID-19 deaths that we identified among working decedents may have resulted from transmission of infection in the workplace, at home, or elsewhere in the community. Yet, there is reason to think that work contributed to the high COVID-19 mortality rates in certain occupational groups. The occupations with highest mortality in California overlap with the critical infrastructure sectors that were exempted from that state’s stay-at-home order [5]. These occupations tend to involve manual labor or in-person provision of services that cannot be done remotely, and many require proximity to others at work [44, 45]. Furthermore, inadequate access in the workplace to exposure controls including ventilation and personal protective equipment may be contributory. One study found strong correlations between worker complaints to federal and state OSHA that raise concerns about workplace conditions and exposure to SARS-CoV-2 and subsequent COVID-19 cases and deaths [46]. Even the occupations that we generally associate with outdoor work, like agriculture and construction, can put workers into close contact within poorly ventilated spaces, such as in produce packing sheds, indoor construction sites, and vehicles. Furthermore, a study of agricultural workers found working outdoors and working in the fields to be risk factors for SARS-CoV-2 infection, perhaps because a lower perceived risk of outdoor work affected behavior or practices [47]. Thus, the conditions of work in these high-mortality occupations may have created opportunities for workplace exposure to SARS-CoV-2. Workplace outbreaks provide further evidence. In Los Angeles County, nearly 60% of non-residential, non-healthcare workplace outbreaks that occurred through September 30, 2020 were in industrial sectors related to many of the occupations that we identified as having elevated mortality: manufacturing, retail trade, and transportation and warehousing [10]. In Toronto, Canada, a similar analysis through June 2020 found the majority (68%) of workplace outbreaks in manufacturing, agriculture, and transportation and warehousing [48]. In Europe, analysis of outbreak data from March through July 2020 revealed large numbers of clusters in the food packaging and processing sectors, in factories and manufacturing, and in office settings [49].

This study has several limitations. Our definition of COVID-19 fatality required a positive polymerase chain reaction (PCR) test result for SARS-CoV-2. Thus, COVID-19 decedents in California who did not undergo PCR testing were excluded, potentially underestimating the occupational burden of COVID-19 deaths. However, in light of the substantial increases in other causes of death in California in 2020 [50], our use of a specific definition of COVID-19 fatality had advantages over analyses of all-cause mortality [11]. Further study of any differential impact of defining COVID-19 fatality using the COVID-19 ICD-10 code would be useful, although the comparability of our findings and those of the study of Massachusetts deaths that used ICD-10 is reassuring [17]. The working status of some decedents may have been misclassified, such as unemployed decedents classified as likely working. Despite this possible misclassification, matching to state employment records to confirm working status is, to our knowledge, unique among California mortality studies and represents an advance that can be applied to other occupational diseases in the future. Another limitation is that our use of “usual occupation” listed on the death certificate may have misclassified the occupation of some decedents who were working in a different occupation at the time of infection. In addition, we used ACS data from 2019, as 2020 data were not yet available. Pandemic-related changes in employment that occurred in California in 2020 may mean that denominators were overestimated [51]. As a result, we may have underestimated the COVID-19 mortality rates for some occupations. Finally, by focusing on working-age decedents, we did not address the potential burden of COVID-19 on workers 65 years of age and older. Nonetheless, given their lower participation in the labor force, our decision to exclude older decedents undoubtedly reduced misclassification among the likely working group.

Our findings have implications for prevention. Given the likelihood that COVID-19 fatalities among working Californians included work-related cases, the occupations with elevated mortality rates should be prioritized by public health and regulatory authorities to ensure that non-pharmaceutical interventions, such as use of respiratory protection or face coverings, adequate ventilation, and surveillance testing, are implemented in the workplace [37, 52, 53]. These high-mortality occupations also should be highlighted in COVID-19 vaccination campaigns, as they represent populations at high risk of severe outcomes regardless of where transmission occurs. As we plan for future SARS-CoV-2 variants and other respiratory viral pandemics, the occupational burden of COVID-19 mortality in California needs to be considered when crafting policies to mitigate disease transmission, including early implementation of non-pharmaceutical interventions in high-mortality occupations, rapid deployment of wage replacement programs that would allow those at high risk of poor outcomes to avoid in-person work, and prioritization of workers in high-mortality occupations for testing and vaccination when available [46, 54]. More broadly, future pandemic responses must consider the concentration of historically disadvantaged racial/ethnic minorities, including undocumented immigrants, in high mortality occupations.
